# Non-Toxin-Producing *Bacillus cereus* Strains Belonging to the *B. anthracis* Clade Isolated from the International Space Station

**DOI:** 10.1128/mSystems.00021-17

**Published:** 2017-06-27

**Authors:** Kasthuri Venkateswaran, Nitin K. Singh, Aleksandra Checinska Sielaff, Robert K. Pope, Nicholas H. Bergman, Sandra P. van Tongeren, Nisha B. Patel, Paul A. Lawson, Masataka Satomi, Charles H. D. Williamson, Jason W. Sahl, Paul Keim, Duane Pierson, Jay Perry

**Affiliations:** a Biotechnology and Planetary Protection Group, Jet Propulsion Laboratory, California Institute of Technology, Pasadena, California, USA; b National Biodefense Analysis and Countermeasures Center, Ft. Detrick, Maryland, USA; c Department of Medical Microbiology, University of Groningen, University Medical Center Groningen, Groningen, The Netherlands; d Department of Microbiology and Plant Biology, University of Oklahoma, Norman, Oklahoma, USA; e National Research Institute of Fisheries Science, Japan Fisheries Research and Education Agency, Kanagawa, Japan; f The Pathogen and Microbiome Institute, Northern Arizona University, Flagstaff, Arizona, USA; g Johnson Space Center, Houston, Texas, USA; h Marshall Space Flight Center, Huntsville, Alabama, USA; University of California, Riverside

**Keywords:** *Bacillus*, *Bacillus anthracis*, *Bacillus cereus*, genomics, ISS, spores

## Abstract

In an ongoing Microbial Observatory investigation of the International Space Station (ISS), 11 *Bacillus* strains (2 from the Kibo Japanese experimental module, 4 from the U.S. segment, and 5 from the Russian module) were isolated and their whole genomes were sequenced. A comparative analysis of the 16S rRNA gene sequences of these isolates showed the highest similarity (>99%) to the *Bacillus anthracis*-*B. cereus*-*B. thuringiensis* group. The fatty acid composition, polar lipid profile, peptidoglycan type, and matrix-assisted laser desorption ionization–time of flight profiles were consistent with the *B. cereus sensu lato* group. The phenotypic traits such as motile rods, enterotoxin production, lack of capsule, and resistance to gamma phage/penicillin observed in ISS isolates were not characteristics of *B. anthracis*. Whole-genome sequence characterizations showed that ISS strains had the *plcR* non-*B. anthracis* ancestral “C” allele and lacked anthrax toxin-encoding plasmids pXO1 and pXO2, excluding their identification as *B. anthracis*. The genetic identities of all 11 ISS isolates characterized via *gyrB* analyses arbitrarily identified them as members of the *B. cereus* group, but traditional DNA-DNA hybridization (DDH) showed that the ISS isolates are similar to *B. anthracis* (88% to 90%) but distant from the *B. cereus* (42%) and *B. thuringiensis* (48%) type strains. The DDH results were supported by average nucleotide identity (>98.5%) and digital DDH (>86%) analyses. However, the collective phenotypic traits and genomic evidence were the reasons to exclude the ISS isolates from *B. anthracis*. Nevertheless, multilocus sequence typing and whole-genome single nucleotide polymorphism analyses placed these isolates in a clade that is distinct from previously described members of the *B. cereus sensu lato* group but closely related to *B. anthracis*.

**IMPORTANCE** The International Space Station Microbial Observatory (Microbial Tracking-1) study is generating a microbial census of the space station’s surfaces and atmosphere by using advanced molecular microbial community analysis techniques supported by traditional culture-based methods and modern bioinformatic computational modeling. This approach will lead to long-term, multigenerational studies of microbial population dynamics in a closed environment and address key questions, including whether microgravity influences the evolution and genetic modification of microorganisms. The spore-forming *Bacillus cereus sensu lato* group consists of pathogenic (*B. anthracis*), food poisoning (*B. cereus*), and biotechnologically useful (*B. thuringiensis*) microorganisms; their presence in a closed system such as the ISS might be a concern for the health of crew members. A detailed characterization of these potential pathogens would lead to the development of suitable countermeasures that are needed for long-term future missions and a better understanding of microorganisms associated with space missions.

## Abstract (precis)

The International Space Station Microbial Observatory (Microbial Tracking-1) study is generating a microbial census of the space station’s surfaces and atmosphere by using advanced molecular microbial community analysis techniques supported by traditional culture-based methods and modern bioinformatic computational modeling. This approach will lead to long-term, multigenerational studies of microbial population dynamics in a closed environment and address key questions, including whether microgravity influences the evolution and genetic modification of microorganisms. The spore-forming *Bacillus cereus sensu lato* group consists of pathogenic (*B. anthracis*), food poisoning (*B. cereus*), and biotechnologically useful (*B. thuringiensis*) microorganisms; their presence in a closed system such as the ISS might be a concern for the health of crew members. A detailed characterization of these potential pathogens would lead to the development of suitable countermeasures that are needed for long-term future missions and a better understanding of microorganisms associated with space missions.

## INTRODUCTION

The *Bacillus* genus, almost by default, is the phenotypic depository for a growing collection of Gram stain-positive, fermentative, aerobic, and spore-forming rods. The taxonomy of the genus *Bacillus* is in a state of flux because of phenotypic changes related to various stressors (1, 2). Molecular methods, particularly 16S rRNA gene analysis, have contributed tremendously to the description of novel taxa close to the genus *Bacillus*, as well as the reclassification of many species previously incorrectly placed within this genus (3). Noteworthy with respect to this study is the fact that several novel *Bacillus* species and genera were also identified in the National Aeronautical and Space Administration (NASA) clean rooms where spacecraft are assembled (4–11).

Several members of the *Bacillus cereus* group of bacteria demonstrating widely different phenotypes (12, 13) were also isolated from NASA clean room surfaces, including several isolates that could not be distinguished from *B. anthracis* by low-resolution molecular typing methods (14). The *B. cereus sensu lato* group defines the collective epitome of closely related species. It consists of species with high medical (emetic toxin; *B. cereus*), biowarfare (virulence plasmids; *B. anthracis*), and economic (biological insecticides; *B. thuringiensis*) importance (15). In addition to these species, *B. cytotoxicus*, *B. mycoides*, *B. pseudomycoides*, *B. toyonensis*, and *B. weihenstephanensis* were recently included in the *B. cereus* group (13, 16). Being in a subdivision of the genus *Bacillus*, members the *B. cereus* group show a high level of genetic relatedness and have been advocated to be one species (1,2). The situation for the delineation of *B. anthracis* and *B. anthracis*-like *B. cereus* is even more complicated (17, 18). General guidelines have been proposed, but they fall short for taxonomic resolution (19). In contrast to the differences in phenotypes, Helgason et al. (12) showed that *B. anthracis* should be considered a lineage of *B. cereus* on the basis of multilocus sequence typing (MLST) by analyzing nine chromosomal genes. When toxin-producing *B. anthracis* strains are considered *B. cereus* on the basis of genomic characteristics, such a taxonomic establishment will have ramifications for its virulence and the potential to transfer genes horizontally within the *B. cereus* group (12, 20).

Okinaka et al. (19) raised the fundamental question of “anthrax, but not *B. anthracis*” and further questioned what constitutes *B. anthracis*. Should this species be classified strictly on the basis of the definition of clinical disease or on the basis of another phenotype? The United States Centers for Disease Control and Prevention expanded the list of phenotypic and genotypic properties of *B. anthracis* to define it strictly as capsule producing, nonmotile, nonhemolytic, susceptible to gamma phage, susceptible to penicillin, and having other cell wall, capsule, and 16S RNA features (17). Furthermore, the *B. anthracis plcR* gene has a nonsense mutation that serves as a genotype marker (21). Test results of *B. anthracis* isolates indicated that this nonsense mutation was present in all 89 *B. anthracis* isolates but was not present in a variety of *B. cereus* relatives (22).

In an ongoing effort of the International Space Station (ISS) Microbial Observatory investigation, NASA is cataloging the total and viable microbial communities of crew-associated environments to a degree that was not previously possible (23, 24). These cataloging efforts have produced vast amounts of data stemming from several automated state-of-the-art molecular methods. Molecular analyses of hundreds of *Bacillus* cultures isolated from the ISS revealed the presence of 11 strains belonging to the *B. cereus* group of bacteria in various modules of the ISS. These strains were isolated during different expeditions not only from U.S. segment Harmony Node 2 of the ISS (this study) but also from the Kibo Japanese experimental module (JEM; NASA archived culture collection) and the Russian segment Zvezda Service Module (DOS-8) (25).

Because the initial molecular screening results led researchers to suspect that these strains were *B. anthracis*, detailed phenotypic, genotypic, and toxigenic characterizations were warranted in addition to the traditional microbiological characterization. The reduced cost of genomic sequencing allows for polyphasic taxonomy, integrating whole-genome sequencing (WGS) information and matrix-assisted laser desorption ionization–time of flight (MALDI-TOF) spectra with the main phenotypic characteristics to define new bacterial taxa (26).

## RESULTS AND DISCUSSION

**FIG 1:**
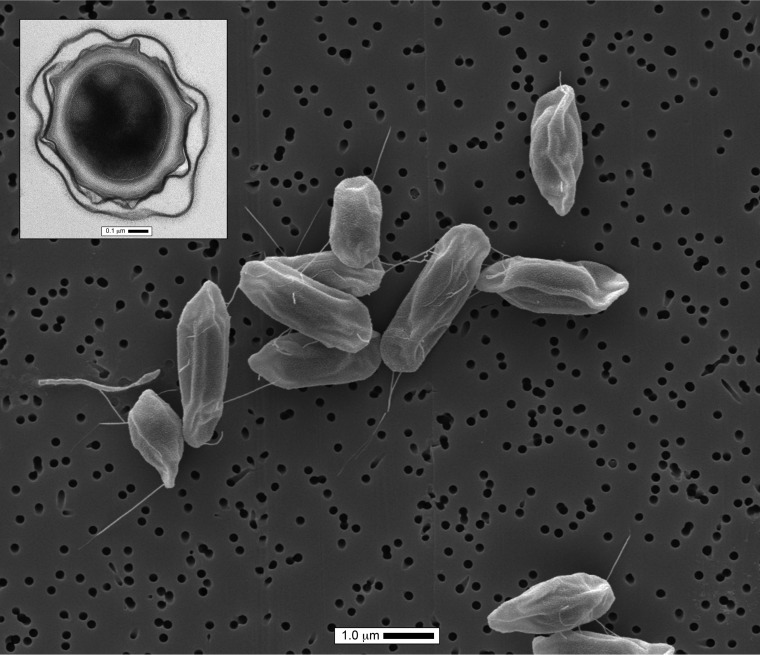
Electron micrographs of ISSFR-003 spores. The inset is a transmission electron micrograph exhibiting the exosporium characteristics of the *B. cereus* group of species. The scanning electron micrograph clearly shows the presence of pili that were also reported in some strains of *B. cereus*.

Electron microscopy images showed that these spores are typical of the *B. cereus* group (Fig. 1). The characteristic spore structure with an exosporium is seen in the transmission electron microscopy (TEM) cross-section inset. The longitudinal scanning electron microscopic (SEM) image of spores reveals an extended loose exosporium with appendages, as has been previously described for some *B. cereus* and *B. thuringiensis* isolates (27–29). The absence of such appendages in *B. anthracis* suggests that the ISS isolates were not *B. anthracis*.

### Phenotypic characterization.

All United States and Japanese isolates produced colonies that were gray and beta-hemolytic with a regular edge and a ground-glass-like appearance typical of *B. cereus* strains. India ink staining indicated that they all lacked a capsule. The ISS isolates were motile and resistant to both gamma phage and penicillin. Likewise, all five Russian isolates were phenotypically characterized as described elsewhere and exhibited characteristics similar to those of the United States and Japanese isolates (25). Cells are Gram stain-positive, spore-forming, aerobic, and motile rods (1.2 to 1.5 by 2 to 5 μm). Spores have an extended loose exosporium with appendages. The colony morphology, beta-hemolytic activity, motile nature, resistance to penicillin as well as gamma phage, and absence of a capsule are properties consistent with what is typically found in non-*B. anthracis* members of the *B. cereus* group.

All 11 strains were biochemically characterized with bacillus-specific test strips, and the results of the tests are shown in Table 1. Briefly, all ISS isolates were identified as members of the *B. cereus* group (00060013 profile, according to Microgen Bioproducts software). The ISS isolates failed to break down mannose as *B. anthracis* Ames and *B. thuringiensis* strains do. Similarly, salicin was not assimilated by both *B. anthracis* Ames and ISS isolates but this sugar was utilized and acid was produced by *B. cereus* and *B. thuringiensis*. Likewise, citrate was not utilized as a sole carbon source by *B. anthracis* Ames and ISS isolates. Differential phenotypic characteristics, as noticed in OmniLog profiling, are shown in Table S1 in the supplemental material and compared with those of *B. anthracis* Ames. Unlike *B. anthracis*, ISS isolates did not utilize mannose, *N*-acetyl-d-galactosamine, methyl pyruvate, d-malic acid, or lithium chloride as a sole carbon source. However, ISS isolates assimilated d-trehalose, sucrose, *N*-acetyl-d-glucosamine, 1% sodium lactate, d-glucose-6-PO_4_, l-serine, d-gluconic acid, and β-hydroxy-D, l-butyric acid, whereas *B. anthracis* Ames did not.

10.1128/mSystems.00021-17.3 TABLE S1  Differential phenotypic characteristics of strain ISSFR-3F and *B. anthracis* Ames. Download TABLE S1, DOCX file, 0.01 MB. Copyright © 2017 Venkateswaran et al. 2017 Venkateswaran et al. This content is distributed under the terms of the Creative Commons Attribution 4.0 International license.

**TABLE 1:** Biochemical test results showing differential characteristic features of strains in the *B. cereus* group^a^

Substrate	1^b^	2	3	4	5	6	7	8	9
Cellobiose	−	−	+	+	+ (w)^c^	+ (w)	−	−	+
Mannose	−	**+**	−	+	+	+	+	−	−
Salicin	−	−	+	+	+	+	+	+	+
Sucrose	+	+	+	+	+	−	−	+	−
Trehalose	+	+	+	+	+	+	+	+	−
Methyl-d-glucoside	−	−	−	−	−	−	−	+	−
Arginine dihydrolase	+	+	+	+	−	+	+	+	−
Citrate utilization	−	−	+	+	−	−	−	+	−

a Strains: 1, this study (11 strains); 2, *B. anthracis* Ames; 3, *B. cereus* CECT 148^T^; 4, *B. thuringiensis* CECT 197^T^; 5, *B. mycoides* CECT 4128^T^; 6, *B. weihenstephanensis* LMG 18989^T^; 7, *B. pseudomycoides* CECT 7065^T^; 8, *B. toyonensis* BCT-7112^T^; 9, *B. cytotoxicus* NVH 391-98^T^.

b Strain 1 is from the present study, and the results for other strains are from reference 11. All strains did not produce acid from arabinose, mannitol, inositol, raffinose, rhamnose, sorbitol, xylose, adonitol, or galactose; did not utilize methyl-d-mannoside, inulin, or melezitose as a sole carbon source; did not produce indole or β-galactosidase; did not reduce nitrate to nitrite; and were positive for the Voges-Proskauer reaction.

c w, weak reaction.

### Chemotaxonomy.

The fatty acid methyl ester (FAME) profile of four representative ISS isolates (ISSFR-3F, JEM-2, S1-R2T1-FB, S1-R3J1-FB-BA1) was similar to that of *B. anthracis* Ames, except for the presence of unsaturated fatty acids such as iso-C_17:1ω10c_ and C_17:0_. The major fatty acids of ISS isolates are iso-C_15:0_, iso-C_16:0_, iso-C_13:0_, iso-C_14:0_, anteiso-C_15:0_, iso-C_17:0_, and C_16:1ω7c/16:1ω6c_. The FAME profiles of all of the type strains in the *B. cereus* group studied were similar (30). However, their FAME profiles also had some quantitative and minor qualitative differences (Table S2). The predominant polar lipids were phosphatidylethanolamine (PE), diphosphatidylglycerol (DPG), phosphatidylglycerol (PG), and unidentified glycolipids, amino lipids, and phospholipids (Fig. S2). The peptidoglycan diamino acid was meso-diaminopimelic acid, which has already been described for species of this group such as *B. cereus*, *B. mycoides*, *B. thuringiensis* (31), *B. toyonensis* (13), and *B. cytotoxicus* (32). Galactose was detected as the whole-cell sugar in ISSFR-3F, JEM-2, S1-R2T1-FB, S1-R3J1-FB-BA1, and *B. anthracis* Ames, while galactose and xylose were detected as the whole-cell sugars in *B. thuringiensis* and *B. cereus*.

10.1128/mSystems.00021-17.4 TABLE S2  Cellular fatty acid compositions of ISSFR-3F, JEM-2, S1-R2T1-FB, S1-R3J1-FB-BA1, and their three closest relatives, *B. thuringiensis*, *B. anthracis* Ames, and *B. cereus*. Download TABLE S2, DOCX file, 0.02 MB. Copyright © 2017 Venkateswaran et al. 2017 Venkateswaran et al. This content is distributed under the terms of the Creative Commons Attribution 4.0 International license.

The MALDI profiles of four representative ISS isolates (ISSFR-3F, JEM-2, S1-R2T1-FB, S1-R3J1-FB-BA1), *B. anthracis* Ames, *B. cereus* ATCC 14579^T^, and *B. thuringiensis* ATCC 10792^T^ were generated with an Ultraflex III instrument. All of the ISS isolates were identified as members of the *B. cereus* group and shared their fingerprint with *B. anthracis* Ames (data not shown). More recently, proteome-based analysis of *B. cereus* group strains revealed a cluster comprising *B. cereus*, *B. anthracis*, *B. thuringiensis*, *B. mycoides*, *B. weihenstephanensis*, and *B. toyonensis*, which have more accurate *m/z* values in common than the remaining species *B. cytotoxicus* and *B. pseudomycoides* (33). Thus, MALDI profiles were not useful for delineation of the members of the *B. cereus* group, as reported here and elsewhere (33).

### Traditional DDH.

The DNA-DNA hybridization (DDH) profiles of four representative ISS isolates (ISSFR-3F, JEM-2, S1-R2T1-FB, S1-R3J1-FB-BA1), *B. anthracis* Sterne, *B. cereus* ATCC 14579^T^, *B. thuringiensis* ATCC 10792^T^, and *B. weihenstephanensis* DSM 11821^T^ were determined. All of the ISS isolates exhibited higher DDH reassociation values with the *B. anthracis* Sterne strain (88 to 90%), suggesting that the ISS isolates belong to the same species when a traditional threshold (>70%) was applied (34, 35). However, it is clear from the DDH results (Table 2) that the ISS isolates are not similar to either the *B. cereus* (42%) or the *B. thuringiensis* (48%) type strain.

**TABLE 2:** Results of traditional DDH

Bacterium^a^ (source)	Strain	Avg % similarity ± SD to labeled DNA from:	
		ISSFR-3F^T^	34F2
*Bacillus* sp. (ISS-U.S.)	ISSFR-3F^T^	100	93.9 ± 4.0
*Bacillus* sp. (ISS-Japan)	JEM-2	88.1 ± 5.3	88.5 ± 7.5
*Bacillus* sp. (ISS-Russia)	S1-R2T1-FB	90.4 ± 2.9	88.6 ± 2.1
*Bacillus* sp. (ISS-Russia)	S2-R3J1-FB-BA1	90.0 ± 5.2	84.8 ± 5.3
*B. anthracis* Sterne	34F2	74.3 ± 0.5	100
*B. cereus*	JCM 2152^T^	41.8 ± 9.9	41.7 ± 6.2
*B. thuringiensis*	IAM 12077^T^	38.8 ± 1.1	40.1 ± 1.2
*B. weihenstephanensis*	DSM 11821^T^	48.0 ± 9.8	36.6 ± 5.7

a *n* = 3.

### *B. cereus sensu lato* group of strains selected for genomic analysis.

In addition to 11 ISS strains, isolates belonging to *B. anthracis* (7 strains), *B. cereus* (3 strains), *B. cytotoxicus* NVH391 98, *B. mycoides* ATCC 6462, *B. pseudomycoides* DSM 12442, *B. thuringiensis* ATCC 10792, *B. toyonensis* BCT 7112, *B. wiedmannii* FSLW8 0169, and *B. weihenstephanensis* DSM 11821 were included in all of the genomic analyses described below. Type strains, near completion of the genome, and low contig numbers were the criteria to select the genomes and comparative analyses performed. In addition to *B. anthracis* Ames, six other isolates bearing at least one virulence plasmid were included. *B. cereus* biovar *anthracis* strain CI was also compared because this strain possesses both plasmids but is not considered *B. anthracis* (36). Furthermore, *B. cereus* AH820, a non-plasmid-carrying strain that was isolated from a patient and reported to be a closer relative to *B. anthracis* but still forms a different phylogenetic clade (37), was added to this comparative genomic analysis.

### dDDH.

The results of digital DDH (dDDH) showed >86.2% similarity to the *B. anthracis* Ames genome for all 11 strains (Table 3). A similarity of >70% indicates that the query species is the same as a reference species on the basis of the recommended intergenomic distance formula calculation (38). However, the similarities between the ISS isolates and type strains *B. cereus* ATCC 14579^T^, *B. cytotoxicus* NVH391 98^T^, *B. mycoides* ATCC 6462^T^, *B. pseudomycoides* DSM 12442^T^, *B. thuringiensis* ATCC 10792^T^, *B. toyonensis* BCT 7112^T^, and *B. weihenstephanensis* DSM 11821^T^ were <46.6% (Table 3). On the basis of the dDDH analyses, it is confirmed that the ISS isolates are not close relatives of either the *B. cereus* or the *B. thuringiensis* type strain.

**TABLE 3:** Results of dDDH^a^

Query	Intergenomic distance (%)							
	*B. anthracis* Ames	*B. cereus* ATCC 14579	*B. cytotoxicus* NVH391 98	*B. mycoides* ATCC 6462	*B. pseudomycoides* DSM 12442	*B. thuringiensis* ATCC 10792	*B. toyonensis* BCT 7112	*B. weihenstephanensis* DSM 11821
ISSFR-3F	88.1	45.1	26.1	38.6	27.3	44.2	43.3	38.5
ISSFR-9F	88.1	45.1	26.1	38.6	27.3	44.2	43.3	38.4
ISSFR-23F	86.9	44.7	26.1	38.4	27.3	43.9	43.1	38.2
ISSFR-25F	87.2	46.6	28.3	40.3	29.6	46	44.7	40.2
JEM-1	87.9	45.1	26.3	38.7	27.7	44.5	43.3	38.7
JEM-2	88.1	45	26.1	38.5	27.3	44.2	43.3	38.4
S1-R1J2-FB	86.9	44.5	25.7	38.2	27.1	44	42.8	38.2
S1-R2T1-FB	86.8	44.5	25.7	38.2	27.1	44	42.8	38.2
S1-R4H1-FB	87.5	44.7	25.7	38.3	27.1	44.1	42.9	38.3
S1-R5C1-FB	86.2	44.4	25.7	38.2	27.1	43.9	42.8	38.2
S2-R3J1-FB-BA1	87.4	44.6	25.8	38.3	27.2	44	42.9	38.3

a An intergenomic distance of >70% indicates that a query genome is similar to a reference genome.

### Phylogenetic characterization.

**FIG 2:**
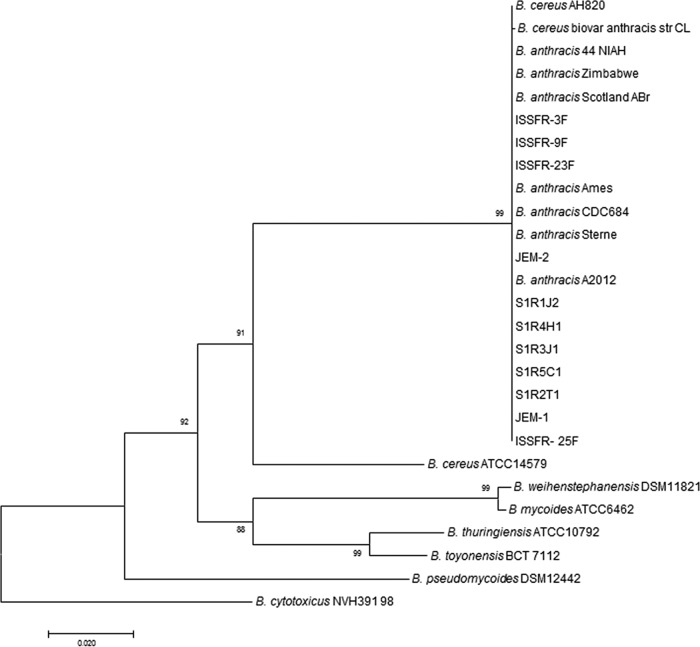
Phylogenetic tree based on GyrB sequences (~1,900 bases) and generated by the neighbor-joining method showing the phylogenetic relationship between 11 ISS isolates and members of the *B. cereus* group. Bootstrap values from 500 replications are shown at branch points. Bar, 0.02 substitution per site.

16S rRNA gene sequencing of all 11 isolates placed them within the *B. cereus* group (Fig. S1). As reported earlier by various groups, our own study confirmed that 16S rRNA gene sequencing analysis could not differentiate these ISS isolates within the *B. cereus* group (23–25). Subsequently, these ISS isolates were phylogenetically characterized by sequencing of the *gyrB* locus, which proved to be more discriminatory than 16S rRNA gene sequencing. The analysis of *gyrB* sequences (Fig. 2) showed that the ISS isolates were most similar to *B. anthracis* (14, 24, 25). However, real-time PCR analysis for specific *B. anthracis* markers such as the *pagA* and *capA* genes (39) did not yield the expected amplicons (264 bp for *capA* and 747 bp for *pagA*). These markers are found on the pXO1 and pXO2 pathogenesis-associated plasmids that are typically carried by *B. anthracis* strains (39). On the basis of the *pag* and *capA* gene analyses, it is confirmed that ISS isolates do not possess these anthrax-associated virulence factors.

10.1128/mSystems.00021-17.1 FIG S1  Phylogenetic tree based on 16S rRNA gene sequences (~1,450 bases) and generated by the neighbor-joining method showing the phylogenetic relationship between ISS isolates and members of the *B. cereus* group. Bootstrap values from 500 replications are shown at branch points. Bar, 0.002 substitution per site. Download FIG S1, TIF file, 0.1 MB. Copyright © 2017 Venkateswaran et al. 2017 Venkateswaran et al. This content is distributed under the terms of the Creative Commons Attribution 4.0 International license.

10.1128/mSystems.00021-17.2 FIG S2  Polar lipid analysis of ISSFR-3F. The predominant polar lipids are PE, DPG, PG, and unidentified glycolipids (GL). Download FIG S2, TIF file, 0.6 MB. Copyright © 2017 Venkateswaran et al. 2017 Venkateswaran et al. This content is distributed under the terms of the Creative Commons Attribution 4.0 International license.

### Whole-genome sequence analysis.

All 11 isolates were screened against the *B. anthracis* genomes contained in RefSeq; however, none of the commonly known *B. anthracis* signature elements were identified. Specifically, all 11 ISS isolates (i) contain the *plcR* ancestral “C” allele that has been used in large-scale phylogenetic analyses to distinguish *B. anthracis* strains from the rest of the *B. cereus* group (22), (ii) lack significant hits to plasmids pXO1 and pXO2, and (iii) are phylogenetically placed outside the *B. anthracis* clade. With respect to plasmid content, all of the ISS isolates contained a 70- to 80-kbp plasmid with closest similarity to the *B. thuringiensis* serovar *konkukian* strain 97-27 plasmid (77 kbp). The ISS isolates were also inspected for *cry* genes (downloaded from Swiss-Prot [40]) common to *B. thuringiensis*; no significant BLAST hits (>70% identity; >200 amino acids; E value, <0.01) to any known *B. thuringiensis cry* genes were found. On the basis of the WGS analyses, it was confirmed that the ISS isolates do not carry key phylogenetically and pathogenically relevant genetic elements reported to be present in *B. anthracis*, *B. cereus*, and *B. thuringiensis*. The DNA G+C content of the ISS strains is 35.4%.

### ANI values.

Subsequently, the assembled genomes of all 11 ISS isolates were subjected to pairwise average nucleotide identity (ANI) analysis against 17 *B. cereus* group members by the algorithm of Goris et al. (41). The ANI values for all of the ISS isolates were >98.5% for *B. anthracis*, whereas the values for other members of *B. cereus* group ranged from 80.9 to 91.5% (Table 4). These results were in concordance with the use of ANI values established by Richter and Rosselló-Móra (42), who set a narrow boundary of ~95 to 96% or more for the same species and further suggested that ANI values could substitute for traditional DDH values. Because the ISS isolates showed ANI values of <91.5% for the *B. cereus* and *B. thuringiensis* genomes, they should not be considered *B. cereus* and *B. thuringiensis* on the basis of these criteria. In a recent polyphasic taxonomic study, despite the fact that a novel species was initially classified as *B. cereus*, on the basis of the ANI value, significant genomic differences (ANI values of <92%) from the members of the *B. cereus* group were reported to allow the strain to be considered *B. toyonensis* (13). ANI calculations were used for pairwise comparisons of the available genomes of the entire *B. cereus* group. Beyond the eight classified species, additional genomospecies that also had ANI values of <94% were detected. In this study, despite the higher ANI values between *B. anthracis* and the ISS isolates that were documented, the absence of pathogenic markers led us to confirm that the ISS strains are divergent from *B. anthracis* but belong to the *B. cereus sensu lato* group.

**TABLE 4:** ANI comparison to genomes from the *B. cereus* group^a^

Query	ANI value (%)							
	*B. anthracis* Ames	*B. cereus* ATCC 14579	*B. cytotoxicus* NVH391_98	*B. mycoides* ATCC 6462	*B. pseudomycoides* DSM12442	*B. thuringiensis* ATCC 10792	*B. toyonensis* BCT 7112	*B. weihenstephanensis* DSM 11821
ISSFR-3F	98.6	91.5	81.3	89.3	81.9	91	90.9	89.1
ISSFR-9F	98.5	91.4	81.3	89.3	81.9	91	90.9	89.1
ISSFR-23F	98.5	91.4	81.3	89.2	82	90.9	90.8	89.05
ISSFR-25F	98.6	91.3	81.1	89.4	82	91.5	91.2	89.2
JEM-1	98.6	91.4	81.4	89.2	82	91.6	90.7	89.1
JEM-2	98.6	91.5	81.3	89.3	81.9	91.3	90.1	89.1
S1-R1J2-FB	98.6	91.4	81.2	89.2	81.9	91	90.9	89.1
S1-R2T1-FB	98.6	91.4	80.9	89.2	82	91	90.9	88.9
S1-R4H1-FB	98.6	91.5	81.2	89.2	81.9	91	90.9	89.1
S1-R5C1-FB	98.6	91.4	81.2	89.2	82	91	91.1	89
S2-R3J1-FB-BA1	98.6	91.4	81.2	89.2	81.9	91	90.9	89.1

a A value of <95% represents a different species.

### MLST analysis.

Even though several phylogenetic analyses suggested that the ISS isolates are *B. anthracis*, the absence of pXO1 and pXO2 biomarkers led us to perform MLST analysis with the *glpF*, *gmk*, *ilvD*, *pta*, *pur*, *pycA*, and *tpi* genes. The genomic contigs of the ISS isolates were searched for the MLST gene sequences, which are standardized for the analysis of the *B. cereus sensu lato* group (43). The sequences retrieved were compared with the sequence types (STs) deposited in the *B. cereus* MLST database (44), concatenated according to the MLST scheme with either the maximum-likelihood or the neighbor-joining algorithm (data not shown). The good congruence between the single-gene reconstructions and the concatenation reinforced the stability of the genealogy observed.

**FIG 3:**
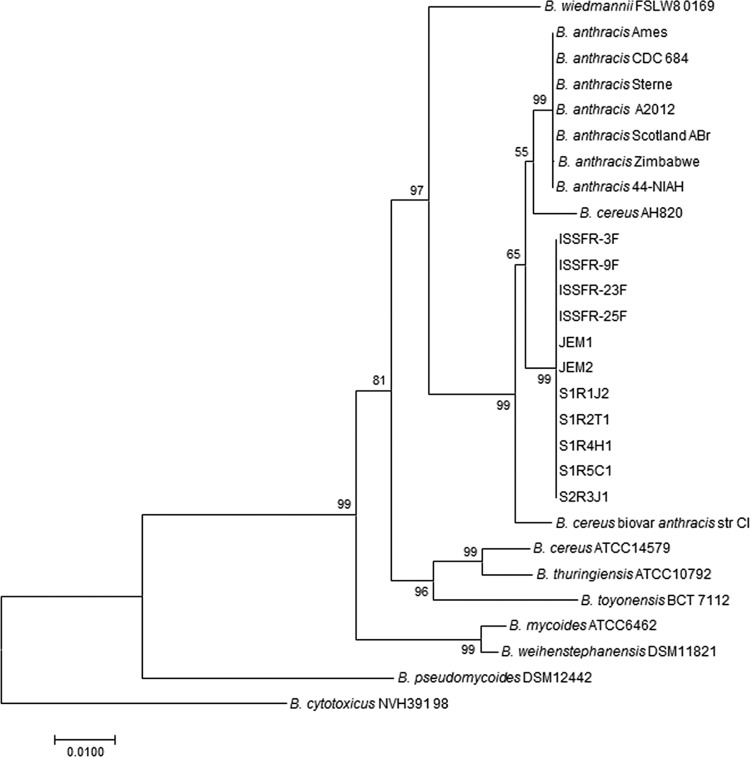
MLST analysis of *Bacillus* strains of this study and related species of the *B. cereus sensu lato* group. The genomic contigs of ISS isolates obtained were searched for *glpF*, *gmk*, *ilvD*, *pta*, *pur*, *pycA*, and *tpi* gene sequences, which are standardized for use in MLST of the *B. cereus sensu lato* group of species (43). The sequences retrieved were compared with the STs deposited in the *B. cereus* MLST database (44), concatenated according to the MLST scheme. It was found that all 11 ST sequences derived from the ISS isolates are identical and do not correspond to any known allele combination. The reconstruction was based on the RAxML algorithm (45), and the bootstrap values were calculated by using 500 replicates. The bar indicates 1% sequence divergence.

It was found that all of these multilocus gene sequences derived from the ISS isolates are identical and do not correspond to any known allele combination. The reconstruction was based on the RAxML algorithm (45). The MLST tree shows that all 11 ISS isolates form a group that is distinct from *B. anthracis* and also do not align with any of the members of *B. cereus* group (Fig. 3). It is evident from this result that the resolution of MLST analysis was higher than that of *gyrB* analysis (Fig. 2). The bootstrap values in Fig. 3 indicate very stable branching, supporting the notion that even though they have ANI and dDDH values in the nondelineating range, the 11 ISS isolates occupy a unique position on the phylogenetic tree. These results reinforce the observation that the ISS isolates represent a new group within *B. cereus sensu lato*.

### Core genome SNP analysis.

**FIG 4:**
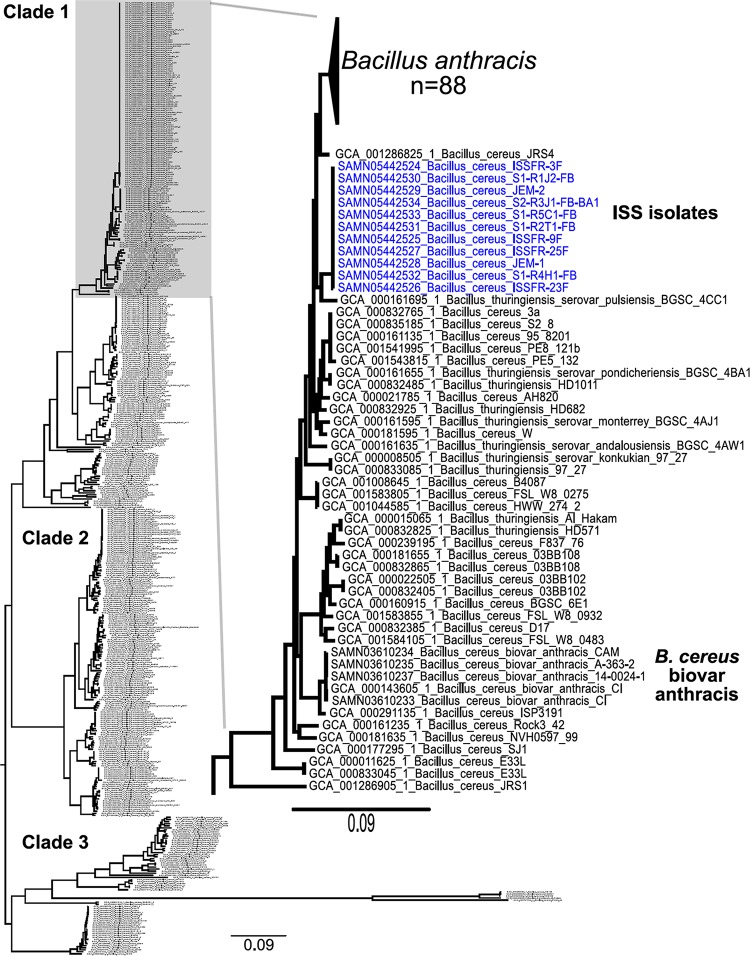
Placement of the ISS isolates into a core genome SNP phylogeny. We used 461 *B. cereus sensu lato* genomes publicly available in the NCBI database to generate a 193,732-character SNP matrix with BWA-MEM and GATK in conjunction with NASP. The tree was inferred with IQ-TREE. The SNPs were called from a 628,820-character core genome alignment. The phylogeny retention index (0.94) and the consistency index (0.31) were calculated with Phangorn. The *B. cereus sensu lato* phylogeny is separated into three major clades. The expanded view on the right provides a closer look at the portion of the tree within clade 1 that includes the ISS isolates. The ISS isolates are closely related to *B. anthracis* but are in a distinct and separate clade.

Relationships among *B. cereus sensu lato* isolates (*n* = 461) were investigated by using a core genome single nucleotide polymorphism (SNP) phylogeny (Fig. 4). The phylogeny was inferred from 193,732 SNPs called from a 628,820-character alignment (core genome). Three major clades can be identified in the phylogeny, which is consistent with previous findings (15, 46). The newly characterized ISS isolates are closely related to *B. anthracis* isolates within clade 1 of the phylogeny. However, the ISS isolates form a clade that is separate and distinct from *B. anthracis*. A core genome SNP analysis including only the ISS isolates indicates that the genomic diversity within the ISS isolates is low. A total of only five SNPs were called from a 4,883,359-character core genome alignment.

In summary, the phenotypic and chemotaxonomic features and generic phylogenetic analyses strongly support the idea that the ISS strains correspond to members of the *B. cereus sensu lato* group but not *B. anthracis*. In contrast, the traditional DDH characterization and high genetic identities of all 11 ISS isolates characterized via *gyrB*, dDDH, and ANI analyses placed them in the same clade as *B. anthracis*. Altogether, the collective phenotypic (motility, positive hemolysis, lack of a capsule, and resistance to gamma phage/penicillin) and genomic (lack of pXO1 and pXO2 plasmids) evidence provides a strong rationale to exclude the ISS isolates from *B. anthracis*. Finally, the MLST and whole-genome SNP analyses placed these isolates in a clade that, while related, is distinct from *B. anthracis* and from previously described members of the *B. cereus sensu lato* group.

## MATERIALS AND METHODS.

### Strains and sampling locations.

Eleven *Bacillus* strains were isolated from various locations of the ISS interior during several missions as shown in Table 5. Brief descriptions of three different ISS module samplings that exhibited incidence of the *B. cereus sensu lato* group of species are shown below.

**TABLE 5:** Sample collection characteristics of *Bacillus* strains isolated from various surfaces of ISS modules

Strain	Collection date (mo/day/yr)	Sample type	Source	ISS location	Returned via STS/expedition
ISSFR-3F	5/1/11	Air	40-mo-old HEPA filter	U.S. Node 2	STS-134/ULF6
ISSFR-9F	5/1/11	Air	40-mo-old HEPA filter	U.S. Node 2	STS-134/ULF6
ISSFR-23F	5/1/11	Air	40-mo-old HEPA filter	U.S. Node 2	STS-134/ULF6
ISSFR-25F	5/1/11	Air	40-mo-old HEPA filter	U.S. Node 2	STS-134/ULF6
JEM-1	4/30/09	Surface	Air diffuser, overhead 8 aft	Kibo JEM	Expedition 19
JEM-2	4/30/09	Surface	Air diffuser, overhead 8 aft	Kibo JEM	Expedition 19
S1-R1J2-FB	4/28/04	Surface	Walls, tables, and toilet areas, communication panel surface around buttons	Russian Zvezda service module	Soyuz TMA-3/expedition 8, Delta Mission
S1-R2T1-FB	4/28/04	Surface	Walls, tables, and toilet areas, table surface	Russian Zvezda service module	Soyuz TMA-3/expedition 8, Delta Mission
S1-R4H1-FB	4/28/04	Surface	Walls, tables, and toilet areas, handlebar of toilet door/outer side of urine collector	Russian Zvezda service module	Soyuz TMA-3/expedition 8, Delta Mission
S1-R5C1-FB	4/28/04	Surface	Walls, tables, and toilet areas, around nozzle of warm-water container	Russian Zvezda service module	Soyuz TMA-3/expedition 8, Delta Mission
S2-R3J1-FB-BA1	10/10/05	Surface	Walls, tables, and toilet areas, joint between wall panels 416 and 417	Russian Zvezda service module	Soyuz TMA-6/expedition 11

### U.S. laboratory Harmony Node 2 BFEs.

Twenty-one filter elements known as bacterial filter elements (BFEs) were distributed throughout the ISS in several U.S. segment modules. Each BFE consists of a 20-mesh prefilter debris screen and 10.2-cm-deep nonwoven borosilicate, pleated, high-efficiency particulate arrestance (HEPA) filter medium. The pleated HEPA filter elements aboard the ISS are a component of the cabin ventilation system that includes a heat exchange process to control humidity. Astronauts replace the BFEs on a scheduled maintenance cycle ranging from 2.5 to 5 years, depending on the location. The European-provided Columbus laboratory module and the Japanese-provided Kibo laboratory module also incorporate HEPA-rated filter elements of a different design in their respective ventilation systems. The cabin environment is typically maintained between 35 and 45% relative humidity and 22 and 24°C. A detailed microbiological characterization of the HEPA filter system examined in this study was presented elsewhere (23). The particulate materials collected from ISS BFE serial number 0049 were designated the ISS HEPA samples during this study. This particular filter element was in service aboard the ISS in the Harmony Node 2 module for approximately 40 months.

A number of microbiological and molecular techniques were used to analyze the ISS HEPA samples to reveal the makeup of cultivable, viable, and total microorganisms. The HEPA medium of the BFEs was divided into small pieces, and then sterile scalpels were used to aseptically collect particulates from these pieces for quantitative measurements. Approximately 1 g of particles collected from the HEPA medium was weighed, placed into a sterile tube with 25 ml of sterile phosphate-buffered saline (PBS), and vortexed for 1 min. Large particles were allowed to settle after vigorous mixing. Aliquots of samples were carefully siphoned, 1 ml was allocated for culture-based analysis, and 15 ml was allocated for culture-independent analysis. After serial 10-fold dilution in sterile PBS, 100 μl of the sample suspension was spread onto two plates of R2A medium (BD Difco, Franklin Lakes, NJ) and incubated at 25°C for 2 to 7 days to estimate bacterial populations. Bacterial density was determined by counting the bacterial CFUs per gram of material. Four strains were placed within the *B. cereus* group (23) and assigned designations with the prefix ISSFR after the phylogenetic characterization of hundreds of strains.

### Kibo JEM.

The surface sampler kit (SSK) was used to collect microbial samples from surfaces in the JEM of the ISS. The SSK is packaged in a Nomex pouch. A pair of scissors is tethered to the inside of the pouch cover, and the pouch holds a Sharpie marker pen, a colony density chart for in-flight visual analysis of colony growth on the slides, and 10 sampling packets. Each sampling packet contains two Biotest Hycon Contact Slides (one Trypticase soy agar [TSA] medium slide and one rose bengal agar [RB] medium slide), one tube of 0.5% phosphate buffer, one tube of freezer medium with an external tube, two biological specimen bags, and a biocide wipe/towelette. The slide medium includes a transparent cover and a slide seal. A label is affixed to each slide to record the date, sampling site, and visual analysis results. Samples collected from the air diffuser, overhead 8 aft of the Kibo JEM with the SSK in Expedition 19 were assayed at the Johnson Space Center microbiology laboratory (Houston, TX) after return to Earth. All bacterial colonies grown on TSA were purified and archived for further studies. When the 91 archived strains were phylogenetically characterized, 2 were placed within the *B. cereus* group. These strains were assigned designations with the prefix JEM.

### Russian Zvezda service module.

Samples were collected from various locations in the human living quarters of the Russian Zvezda service module (DOS-8) of the ISS to investigate the bacterial microbiota. Samples were collected from the Russian segment during several spaceflight missions as part of a microgravity applications program (MAP) of the European Space Agency and the SAMPLE experiments (47). The sampling system consisted of a swab rinse kit (927C SRK) tube without medium containing a Dacron applicator (Copan Italia SPA Diagnostics Inc., Brescia, Italy). Swabs were premoistened with 55 µl of sterile, clinical-grade 0.9% PBS (B. Braun Melsungen AG, Melsungen, Germany) by DNA-free techniques and sent to the ISS as part of a sample collection kit payload. After collection, samples were stored and transported to the microbiology laboratory via cold pack (4 ± 2°C) to the microbiology laboratory at the University Medical Center, Groningen, the Netherlands, for subsequent processing. The samples were suspended in fastidious broth (FB; Mediaproducts BV, Groningen, the Netherlands) and incubated for 1 to 2 days at 37°C. The suspension was then plated on blood agar (Mediaproducts BV) and incubated for another 1 to 2 days at 37°C. Pure cultures of isolates were maintained in Microbank tubes (Pro-Lab Diagnostics, Richmond Hill, ON, Canada) at −80°C. When required, the samples were further cultivated overnight on blood agar plates at 37°C for various analyses. When the bacterial strains isolated were phylogenetically characterized to the species level, five strains could only be identified as nonvirulent *B. cereus/ B. anthracis sensu lato*, as described previously (25). These strains were assigned designations with a prefix of either S1 or S2.

### Phenotypic characterization.

The United States and Japanese isolates were plated onto 5% sheep blood agar plates (Hardy Diagnostics, Santa Maria, CA) and incubated at 35°C for 22 h. Gram staining of a representative colony of each isolate was performed by standard procedures (48). Capsule production was assayed following plating on bicarbonate medium and incubation with 15% CO_2_ at 35°C for approximately 24 h (49). Isolates were further assayed for motility (50), gamma phage susceptibility (51), and penicillin sensitivity (52). The Russian isolates were characterized, and the results were published elsewhere (25).

The isolates were biochemically identified with a MID-66 Bacillus ID kit (Microgen Bioproducts, Camberley, United Kingdom). The properties tested included fermentation of carbohydrates, indole formation, Voges-Proskauer reaction, nitrate reduction, citrate utilization, and β-galactosidase and arginine dehydrolase activities. The test strip protocol results were analyzed with Microgen ID software (Microgen Bioproducts, Camberley, United Kingdom).

For Biolog carbon substrate utilization profile characterization, a single colony was selected and emulsified in Inoculating Fluid A (Biolog, Hayward, CA) for subsequent inoculation onto the microplate test plate. The ISS isolates, *B. anthracis* Ames, *B. cereus*, and *B. thuringiensis* were cultured on TSA medium in accordance with the manufacturer’s instructions, and bacterial cell suspensions were prepared to a specified transmittance with a turbidimeter as recommended in the user guide. For each isolate, a multichannel pipette was used to inoculate 100 μl of the cell suspension into each well of the microplate and the plate was incubated aerobically at 32°C for 20 h. Microplates were read in the MicroStation semiautomated reader after 20 h, and the identification system’s software (GEN III database, version 5.2.1) interpreted the results (53).

### Sporulation.

Pure cultures of isolate ISSFR-003 grown on nutrient sporulation medium agar plates were removed via washing in 4 ml of sterile filtered water with a spreader bar. Spores were washed three times in ice-cold purified water and allowed to stand for 21 days at 4°C. Samples of the spores were fixed in 4% paraformaldehyde with 1% glutaraldehyde in 0.1 M sodium cacodylate buffer for 4 h. After fixation, the spores were washed to remove fixative and sterility tested. Sterile spore samples were postfixed for 60 min with 1% osmium tetroxide and subsequently stained for 60 min with 0.5% uranyl acetate in water. The samples were then dehydrated through a graded ethanol series, and a portion of each spore sample preparation was saved for SEM processing. The TEM samples were further dehydrated in propylene oxide, infiltrated with Embed812 epoxy resin, and cured at 60°C for 24 h. After curing, the blocks were sectioned (90 to 120 nm), collected on 300-mesh copper grids, and counterstained with 5% uranyl acetate and Reynolds’s lead citrate (54). The TEM sections were imaged in an FEI T12 BioTWIN transmission electron microscope at 100 kV. The SEM samples were critical point dried and coated with gold-palladium prior to imaging on an FEI Quanta 200 FEG scanning electron microscope at 5 kV.

### Chemotaxonomy.

For cellular fatty acids, biomass was harvested from brain heart infusion (BHI) supplemented with 5% defibrinated sheep’s blood agar grown for 24 h at 37°C. FAMEs were extracted with the Sherlock microbial identification system (MIDI Inc.), version 6.1, as described previously (55–57). FAME analysis was carried out with an Agilent Technologies 6890N gas chromatograph, which includes a phenyl methyl silicone fused silica capillary column (HP-2; 25 m by 0.2 mm by 0.33 μm [film thickness]) and a flame ionization detector. Hydrogen was used as the carrier gas. The temperature program was initiated at 170°C and increased by 5°C every minute to a final temperature of 270°C. The relative amount of each fatty acid was expressed in terms of the percentage of total fatty acids.

Polar lipids were extracted in accordance with established procedures via two-dimensional thin-layer chromatography (TLC) on silica gel plates (Macherey-Nagel) (58). Two-dimensional TLC was run with chloroform-methanol-water (65:25:4, vol/vol) as the solvent for the first phase and chloroform-methanol-acetic acid-water (80:12:15:4, vol/vol) as the solvent for the second phase. Phospholipids were identified with molybdenum blue, amino lipids were identified with ninhydrin, glycolipids were identified with alpha-naphthol and molybdophosphoric acid hydrate was used to obtain a total lipid profile (Sigma). Whole-cell sugars were analyzed as previously described (59).

MALDI-TOF mass spectra were obtained with an Ultraflex III instrument operated in linear positive mode under flexControl 3.1 software. External calibration of the mass spectra was done with standard peaks from *Escherichia coli* DH5α (4,346.3, 5,095.8, 5,380.4, 6,254.4, 7,273.5, and 10,299.1 Da). The laser power was set to 120% of the threshold, and five independent spectra comprising 240 laser shots were acquired from each spot. Within an individual spot, the laser was directed manually when required in addition to a predefined lattice raster. Mass spectra were processed with flexAnalysis (version 3.1; Bruker Daltonik) and Biotyper software (version 3.1; Bruker Daltonik).

### DDH.

To extract DNA, cells were cultured in tryptic soy broth supplemented with 2% glycine and cells were harvested by centrifugation after overnight growth. Cell pellets were suspended in Tris-EDTA buffer (pH 8.0) and treated with lysozyme (final concentration, 10 mg/ml) to digest the cell wall. Procedures for extraction of chromosomal DNA and subsequent purification steps were carried out in accordance with standard methods by phenol-chloroform solvent extraction (60, 61). DDH was carried out by microplate hybridization methods (62).

### Targeted gene amplification.

Genomic DNA extraction, amplification, and sequencing of the 16S rRNA gene were performed as described previously (63). Various genetic fragments, such as the 16S rRNA (23), *gyrB* (14), *pag* (39), and *cap* (39) genes, were amplified as established elsewhere, and appropriate positive controls (type strains of *B. cereus* and *B. thuringiensis* and plasmid-carrying *B. anthracis* strains) were included. The 16S rRNA gene-based identification of phylogenetic neighbors was initially carried out against the database of type strains of prokaryotic species with validly published names in EzTaxon-e (64). The *gyrB* gene PCR products were purified and sequenced with the Sanger platform in accordance with a previously established protocol (14). Neighbor-joining phylogenetic analysis was performed with the MEGA software package (65). When the *pag* and *cap* genes of ISS strains were amplified, plasmid-carrying *B. anthracis* strains IP7702 (pXO1 positive) and NMR 162 (pXO2 positive) were also included and confirmation of the appropriate amplicon size was done via agarose gel electrophoresis. The *cap* (forward primer ACT CGT TTT TAA TCA GCC CG and reverse GGT AAC CCT TGT CTT TGA AT) and *pag* (forward primer CAG AAT CAA GTT CCC AGG GG and reverse primer TCG GAT AAG CTG CCA CAA GG) amplifications and PCR conditions were in accordance with established procedures (66).

### Genome sequence analysis.

Among the 11 strains, four United States and two JEM isolates were sequenced on both the Illumina MiSeq and PacBio RSII sequencing platforms. The five Russian isolates were sequenced only by MiSeq. The MiSeq runs yielded, on average, 24 to 54 million 300-bp reads (from 1,402× to 3,093× average coverage), while the PacBio runs yielded 4,000 to 116,000 reads (from 7× to 202× average coverage) (Table 1). Because of the extremely high coverage (>1,000×), Illumina MiSeq reads were randomly downsampled to 100× with an estimated genome size of 5.3 Mbp, resulting in an average of 1.2 to 1.5 million paired-end reads per isolate. Next, the downsampled reads were assembled with iMetAMOS (67) by using IDBA_UD and SPAdes (68). IDBA_UD was selected as the best assembly for all 11 isolates. Low-confidence bases within the selected IDBA_UD (69) assemblies were masked out by mapping all reads to the assembled contigs and detecting conflicting variants with FreeBayes (70). The PacBio reads were assembled as described by Berlin et al. (71). Celera Assembler (72) version 8.3rc1 was used, and PacBio assemblies were polished with Quiver (73); for the PacBio assemblies, a second round of polishing was performed after Quiver with the available MiSeq data as the input to PILON (http://www.broadinstitute.org/software/pilon/).

MLST analysis was carried out as described previously (74). The *Bacillus* MLST scheme employed used seven housekeeping genes, i.e., *glpF* (glycerol uptake facilitator protein), *gmk* (guanylate kinase, putative), *ilvD* (dihydroxy-acid dehydratase), *pta* (phosphate acetyltransferase), *pur* (phosphoribosylaminoimidazolecarboxamide), *pycA* (pyruvate carboxylase), and *tpi* (triosephosphate isomerase) (43).

Pairwise ANI was calculated with an algorithm from Goris et al. (41). dDDH was performed with Genome-to-Genome Distance Calculator 2.0 (GGDC 2.0) (75), which is available at http://ggdc.dsmz.de/. Briefly, the genome sequences in fasta format were submitted to GGDC 2.0 along with the sequences in fasta format for the type strains *B. anthracis* Ames (and six other strains), *B. cereus* biovar *anthracis* CI, *B. cereus* AH820, *B. cereus* ATCC 14579, *B. cytotoxicus* NVH391 98, *B. mycoides* ATCC 6462, *B. pseudomycoides* DSM 12442, *B. thuringiensis* ATCC 10792, *B. toyonensis* BCT 7112, *B. wiedmannii* FSLW8 0169, and *B. weihenstephanensis* DSM 11821. The results were obtained by comparing query genomes (11 isolates) with the reference (17 strains) genomes to calculate intergenomic distances. The results from the recommended calculation formula were chosen as final.

### Whole-genome SNP analysis.

SNPs were called from NUCmer (76, 77) alignments of *B. cereus sensu lato* assemblies to a reference genome (GCA_000008445.1, *B. anthracis* Ames Ancestor A2084) within NASP (78). Duplicated regions of the reference genome were identified with NUCmer and removed from the analysis. Maximum-likelihood phylogenies were inferred from core genome SNPs with IQ-TREE (v1.4.4) (79) by the ultrafast bootstrap method (80) using the GTR+ASC+G4 model. The consistency index and retention index were calculated with Phangorn (81). To ascertain the genomic diversity within the ISS isolates, raw sequencing reads representing nine of the isolates were randomly subsampled to a depth of 3,000,000 reads with the BBTools reformat tool (http://jgi.doe.gov/data-and-tools/bbtools/). The subsampled reads were aligned with the assembly for the JEM-2 isolate with BWA-MEM (82), and SNPs were called by the UnifiedGenotyper method in GATK (83, 84) within NASP. Positions were removed from the analysis if the depth of coverage was <10 or if the proportion of an allele call was <0.9. Trees were viewed in FigTree v1.4.2 (http://tree.bio.ed.ac.uk/software/figtree/), and a figure was generated with InkScape (https://inkscape.org).

### Nucleotide sequence(s).

The whole-genome sequences submitted to the NCBI and NASA GenLab databases were downloaded and characterized during this study. The supporting raw data, scripts, and results are available at ftp://ftp.cbcb.umd.edu/pub/data/issensis. The complete genome sequences were deposited in NCBI as BioProject PRJNA335430 and at the NASA GeneLab system (GLDS-67; https://genelab-data.ndc.nasa.gov/genelab/accession/GLDS-64/).[Supplementary-material tabS2]
