# Supplementary material for: Non-Toxin-Producing Bacillus cereus Strains Belonging to the B. anthracis Clade Isolated from the International Space Station
Source: mSystems. 2017 Jun 27;2(3):e00021-17. doi: 10.1128/mSystems.00021-17 (PMC5487513; doi:10.1128/mSystems.00021-17)
Supplement: TABLE S1 [file sys003172114st1.docx]

**Supplementary Table S1.** Differential phenotypic characteristics between strain

ISSFR-3F and *B. anthracis* Ames*.* All data are from the present study. Both strains are negative for arabinose, cellobiose, inositol, mannitol, rafffinose, rhamnose, sorbitol, xylose, adonitol, galactose, methyl-D-mannoside, inulin, melezitose, indole, β-galactosidase. Using **GEN III MicroPlate ^TM^** the two strains are positive for Dextrin, D-Maltose, D-Cellobiose, Gentiobiose, pH 6, Gelatin, Pectin, L-Lactic Acid, L-Malic Acid, Potassium Tellurite, Tween 40, Acetoacetic Acid, Acetic Acid, Formic Acid, Aztreonam, Sodium Butyrate but negative for pH 5, D-Raffinose, α-D-Lactose, D-Melibiose, β-Methyl-D-Glucoside, D-Salicin, N-Acetyl-β-D-Mannosamine, N-Acetyl Neuraminic Acid, α-D-Glucose, D-Mannose, D-Fructose, D-Galactose, 3-Methyl Glucose, D-Fucose, L-Fucose, L-Rhamnose, Inosine, Fusidic Acid, D-Sorbitol, D-Mannitol, D-Arabitol, myo-Inositol, Glycerol, D-Aspartic Acid, D-Serine, Troleandomycin, Minocycline, Glycyl-L-Proline, L-Alanine, L-Arginine, L-Aspartic Acid, L-Histidine, L-Pyroglutamic Acid, Lincomycin, Niaproof 4, D-Galacturonic Acid, L-Galactonic Acid Lactone, D-Glucuronic Acid, Glucuronamide, Mucic Acid, Quinic Acid, D-Saccharic Acid, Vancomycin, Tetrazolium Violet, Tetrazolium Blue, p-Hydroxy-Phenylacetic Acid, D-Lactic Acid Methyl Ester, Citric Acid, α-Keto-Glutaric Acid, Bromo-Succinic Acid, Nalidixic Acid, α-Keto-Butyric Acid and Sodium Bromate

| Characteristic | ISSFR-3F | *B. anthracis* Ames |
| --- | --- | --- |
| β-hemolytic | P | N |
| Motility | P | N |
| Gamma phage resistance | P | N |
| Penicillin resistance | P | N |
| pXO1 and pXO2 | N | P |
| *cry* gene | N | P |
| *hbl* operon | N | P |
| D-Trehalose | P | N |
| Mannose | N | P |
| Sucrose | P | N |
| N-Acetyl-D-Glucosamine | P | N |
| N-Acetyl-D-Galactosamine | N | P |
| 1% NaCl | P | N |
| 4% NaCl | N | P |
| 8% NaCl | N | P |
| 1% Sodium Lactate | P | N |
| D-Glucose-6-PO4 | P | N |
| L-Serine | P | N |
| D-Gluconic Acid | P | N |
| Methyl Pyruvate | N | P |
| D-Malic Acid | N | P |
| Lithium Chloride | N | P |
| β-Hydroxy-D,L-Butyric Acid | P | N |
| C17:1 iso ω10c | P | N |
| C17:0 | P | N |
