# Supplementary material for: Non-Toxin-Producing Bacillus cereus Strains Belonging to the B. anthracis Clade Isolated from the International Space Station
Source: mSystems. 2017 Jun 27;2(3):e00021-17. doi: 10.1128/mSystems.00021-17 (PMC5487513; doi:10.1128/mSystems.00021-17)
Supplement: TABLE S2 [file sys003172114st2.docx]

**Supplementary Table S2.** Cellular fatty acid compositions of ISSFR-3F ^T^, JEM-2, S1-R2T1-FB, S1-R3J1-FB-BA1 and three closest relatives, *Bacillus* *thuringiensis*, *Bacillus anthracis* Ames*,* and *Bacillus cereus.* Summed feature 2; C_14:0 3OH/16:1 ISO I_ | Summed feature 3; C_16:1 ω7c/16:1 ω6c_ | Summed feature 5; C_18:2 ω6,9c/18:0 ante_ | Summed feature 10; C_18:0/17:0 cyclo_ | Summed feature 11; C_20:0/19:0 cyclo ω8­c_. Strains: 1, ISSFR-3F ^T^; 2, JEM-2; 3, S1-R2T1-FB; 4, S1-R3J1-FB-BA1, 5, *B*. *thuringiensis^T^*; 6, *B. anthracis* Ames; 7, *B. cereus*^T^ CCUG 14579

| Method: QBA1 | BHI + Blood Agar-24 hours at 37 °C; -, not present. All data from this study. Values are percentages of total fatty acids with those representing less than 1% in all strains being omitted.

| Fatty Acid (%) | 1 | 2 | 3 | 4 | 5 | 6 | 7 |
| --- | --- | --- | --- | --- | --- | --- | --- |
| C_10:0_ | - | - | - | - | 2.30 | - | 1.13 |
| C_11:0_ | - | - | - | - | 1.14 | - | 0.30 |
| C_12:0 iso_ | 0.94 | <1 | <1 | <1 | 1.86 | <1 | 0.53 |
| C_12:0_ | - | <1 | <1 | - | 2.72 | - | 1.04 |
| C_13:0 iso_ | 7.25 | 7.65 | 6.08 | 7.28 | 6.40 | 5.81 | 5.38 |
| C_13:0 anteiso_ | 3.33 | 2.65 | 2.35 | 3.10 | 3.45 | 3.73 | 3.09 |
| C_13:0_ | - | - | - | - | 1.28 | - | <1 |
| C_14:0 iso_ | 6.09 | 5.37 | 5.70 | 6.21 | 7.96 | 4.78 | 2.90 |
| C_14:0_ | 3.79 | 3.89 | 3.53 | 3.78 | 5.80 | 3.10 | 4.38 |
| C_15:0 iso_ | **26.24** | **29.04** | **25.59** | **26.83** | **14.67** | **29.71** | **22.72** |
| C_15:0 anteiso_ | 6.08 | 4.44 | 6.31 | 5.40 | 5.60 | 8.94 | 7.14 |
| C_15:0_ | 2.21 | 1.45 | 1.44 | 1.55 | 1.95 | <1 | 1.22 |
| Summed Feature 2 | 2.21 | 2.26 | 2.07 | 2.23 | 1.64 | <1 | 1.87 |
| C_16:0 iso_ | **9.51** | **8.41** | **10.10** | **9.65** | **9.47** | **9.99** | **5.52** |
| Summed Feature 3 | 5.93 | 6.23 | 5.79 | 5.88 | 3.70 | 4.34 | 7.57 |
| C_16:0_ | 5.76 | 5.29 | 6.28 | 5.70 | 9.03 | 8.60 | 6.68 |
| C_17:1 iso ω10c_ | 1.44 | 1.67 | 1.88 | 1.55 | <1 | - | 1.11 |
| C_17:1 iso ω5c_ | 1.59 | 1.93 | 1.67 | 1.58 | <1 | <1 | 2.38 |
| C_17:1 anteiso A_ | <1 | <1 | <1 | <1 | <1 | <1 | 1.64 |
| C_17:0 iso_ | 6.41 | 6.93 | 6.84 | 6.28 | 4.25 | 8.58 | 7.37 |
| C_17:0 anteiso_ | 2.26 | 1.65 | 2.58 | 1.97 | 1.86 | 4.45 | 3.19 |
| C_17:0_ | <1 | <1 | <1 | <1 | 1.48 | -­­­ | <1 |
| Summed feature 5 | 1.23 | 1.03 | 1.39 | 1.19 | <1 | 1.18 | <1 |
| C_18:1 ω9c_ | 1.76 | 1.67 | 1.99 | 1.75 | 1.01 | 1.07 | 1.61 |
| Summed feature 10 | 1.37 | 1.34 | 1.50 | 1.29 | 3.57 | 1.32 | 2.07 |
| C_19:0_ | - | - | - | - | 1.25 | - | <1 |
| Summed feature 11 | - | - | - | - | 2.50 | - | <1 |
