# Supplementary figures and images for: Non-Toxin-Producing Bacillus cereus Strains Belonging to the B. anthracis Clade Isolated from the International Space Station
Source: mSystems. 2017 Jun 27;2(3):e00021-17. doi: 10.1128/mSystems.00021-17 (PMC5487513; doi:10.1128/mSystems.00021-17)

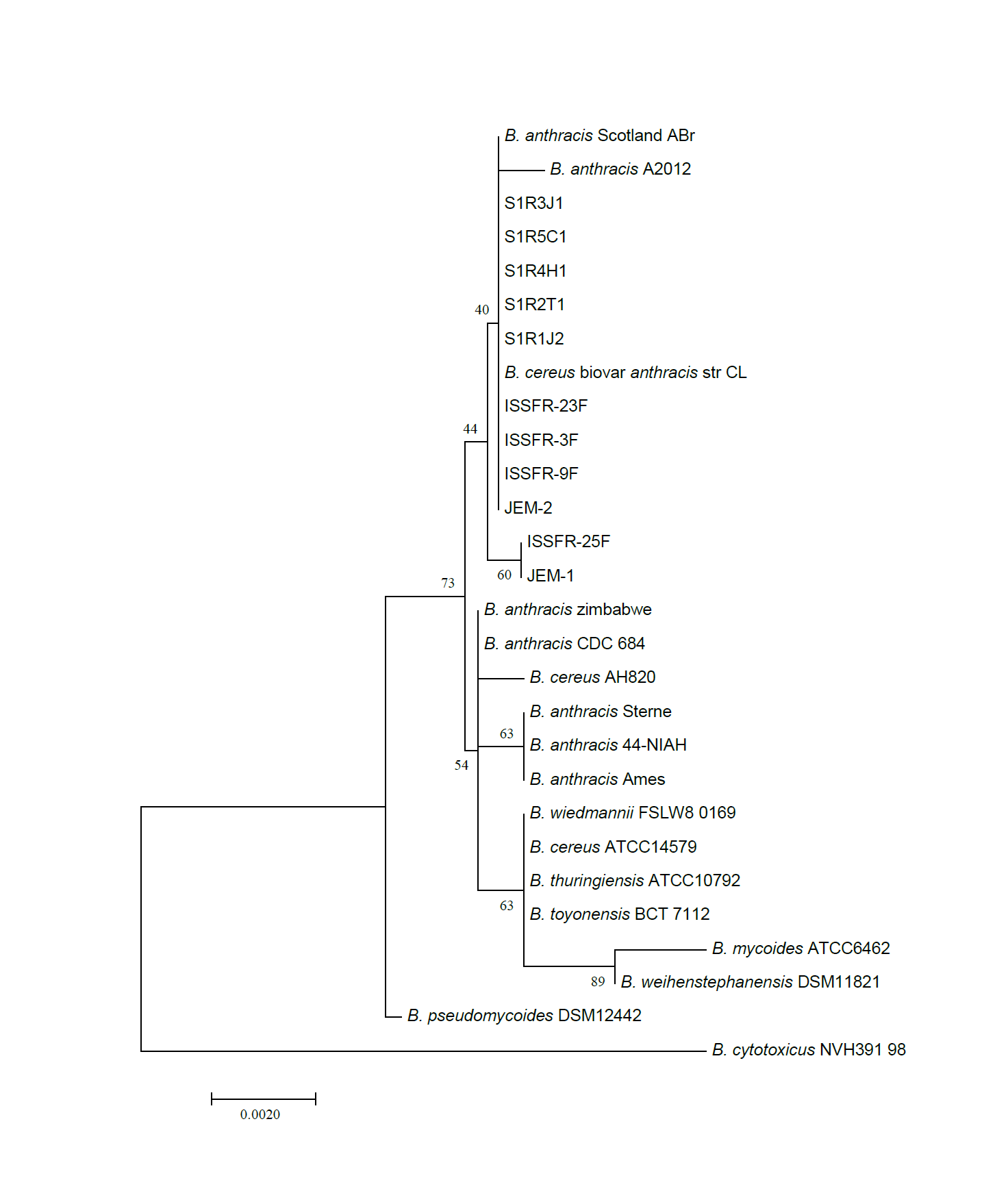

Supplement: FIG S1 [file sys003172114sf3.tif]

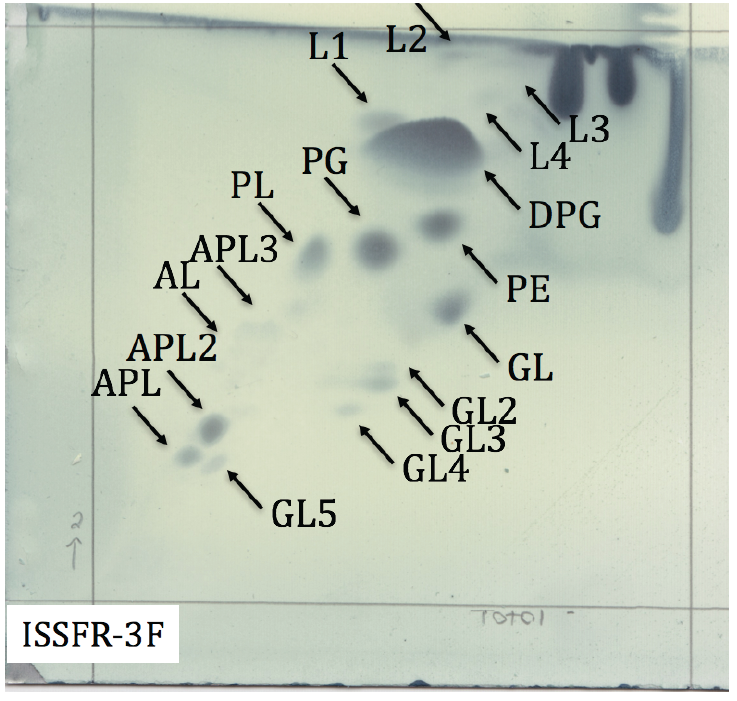

Supplement: FIG S2 [file sys003172114sf4.tif]
